# Supplementary material for: Sustained-input switches for transcription factors and microRNAs are central building blocks of eukaryotic gene circuits
Source: Genome Biol. 2013 Aug 23;14(8):R85. doi: 10.1186/gb-2013-14-8-r85 (PMC4054853; doi:10.1186/gb-2013-14-8-r85)
Supplement: Additional file 5 — HTML Browsable Motif Output. Zipped folder containing all WaRSwap and FANMOD motif output, viewable in a web browser. [file gb-2013-14-8-r85-S5.ZIP › HTML_browsable_motif_output/WaRSwap_ath_tair10_withSelfLoops/sigs_WSWRSLDraw_TAIR10_SLColors-2500.pvals.heatmaps.html/motif_id_14_300031113_tftype_ath_upstream_-3000_0.html]

```
BG_MODEL = WARSwap
MOTIF_ID = 14_300031113
TF_TYPE = ath
UPSTREAM = -3000_0


PVals
FNR = 0.2	FNR = 0.4	FNR = 0.6	FNR = 0.8
deltaG = 60	0.002	0.024	0.056	0.058
deltaG = 70	0.002	0.025	0.056	0.055
deltaG = 80	0	0.03	0.054	0.05

ZScores
FNR = 0.2	FNR = 0.4	FNR = 0.6	FNR = 0.8
deltaG = 60	4.821	2.463	1.755	-0.22
deltaG = 70	4.788	2.444	1.728	-0.214
deltaG = 80	4.86	2.387	1.766	-0.199

StDevs
FNR = 0.2	FNR = 0.4	FNR = 0.6	FNR = 0.8
deltaG = 60	25.385	14.984	3.516	0.342
deltaG = 70	25.514	15.02	3.575	0.318
deltaG = 80	25.299	15.261	3.484	0.342
```
